# In vitro and in vivo anti-malarial activity of plants from the Brazilian Amazon

**DOI:** 10.1186/s12936-015-0999-2

**Published:** 2015-12-18

**Authors:** Renata B. S. Lima, Luiz F. Rocha e Silva, Marcia R. S. Melo, Jaqueline S. Costa, Neila S. Picanço, Emerson S. Lima, Marne C. Vasconcellos, Ana Paula A. Boleti, Jakeline M. P. Santos, Rodrigo C. N. Amorim, Francisco C. M. Chaves, Julia P. Coutinho, Wanderli P. Tadei, Antoniana U. Krettli, Adrian M. Pohlit

**Affiliations:** Laboratório de Princípios Ativos da Amazônia, Coordenação de Tecnologia e Inovação, Instituto Nacional de Pesquisas da Amazônia, Avenida André Araújo, 2936, Petrópolis, 69067-375 Manaus, Amazonas Brazil; Programa de Pós-graduação em Biotecnologia, Universidade Federal do Amazonas, Avenida Gal. Rodrigo Otávio Jordão Ramos, 3000, Coroado I, Campus Universitário, Bloco M, Setor Sul, 69077-000 Manaus, Amazonas Brazil; Centro Universitário do Norte, Rua Dez de Julho, 873, Centro, 69010-060 Manaus, Amazonas Brazil; Escola Superior de Ciências da Saúde, Universidade Estadual do Amazonas, Avenida Carvalho Leal, 1777, Cachoeirinha, 69065-001 Manaus, Amazonas Brazil; Faculdade de Ciências Farmacêuticas, Universidade Federal do Amazonas, Rua Comendador Alexandre Amorim, 330, Aparecida, 69103-000 Manaus, Amazonas Brazil; Embrapa Amazônia Ocidental, Rodovia AM-010, Km 29 (Estrada Manaus/Itacoatiara), Caixa Postal 319, 69010-970 Manaus, Amazonas Brazil; Centro de Pesquisas René Rachou, Fundação Oswaldo Cruz, Avenida Augusto de Lima, 1715, Barro Preto, 30190-002 Belo Horizonte, Minas Gerais Brazil; Laboratório de Malária e Dengue, Coordenação de Sociedade, Ambiente e Saúde, Instituto Nacional de Pesquisas da Amazônia, Avenida André Araújo, 2936, Petrópolis, 69067-375 Manaus, Amazonas Brazil

**Keywords:** *Plasmodium falciparum*, *Plasmodium berghei*, Antiplasmodial, Cytotoxic, *Anacardium occidentale*, *Andropogon leucostachyus*, *Croton cajucara*, *Paullinia cupana*, *Xylopia amazonica*, *Zanthoxylum djalma*-*batistae*

## Abstract

**Background:**

The anti-malarials quinine and artemisinin were isolated from traditionally used plants (*Cinchona* spp. and *Artemisia annua*, respectively). The synthetic quinoline anti-malarials (e.g. chloroquine) and semi-synthetic artemisinin derivatives (e.g. artesunate) were developed based on these natural products. Malaria is endemic to the Amazon region where *Plasmodium falciparum* and *Plasmodium vivax* drug-resistance is of concern. There is an urgent need for new anti-malarials. Traditionally used Amazonian plants may provide new treatments for drug-resistant *P. vivax* and *P. falciparum*. Herein, the in vitro and in vivo antiplasmodial activity and cytotoxicity of medicinal plant extracts were investigated.

**Methods:**

Sixty-nine extracts from 11 plant species were prepared and screened for in vitro activity against *P. falciparum* K1 strain and for cytotoxicity against human fibroblasts and two melanoma cell lines. Median inhibitory concentrations (IC_50_) were established against chloroquine-resistant *P. falciparum* W2 clone using monoclonal anti-HRPII (histidine-rich protein II) antibodies in an enzyme-linked immunosorbent assay. Extracts were evaluated for toxicity against murine macrophages (IC_50_) and selectivity indices (SI) were determined. Three extracts were also evaluated orally in *Plasmodium berghei*-infected mice.

**Results:**

High in vitro antiplasmodial activity (IC_50_ = 6.4–9.9 µg/mL) was observed for *Andropogon leucostachyus* aerial part methanol extracts, *Croton cajucara* red variety leaf chloroform extracts, *Miconia nervosa* leaf methanol extracts, and *Xylopia amazonica* leaf chloroform and branch ethanol extracts. *Paullinia cupana* branch chloroform extracts and *Croton cajucara* red variety leaf ethanol extracts were toxic to fibroblasts and or melanoma cells. *Xylopia amazonica* branch ethanol extracts and *Zanthoxylum djalma*-*batistae* branch chloroform extracts were toxic to macrophages (IC_50_ = 6.9 and 24.7 µg/mL, respectively). *Andropogon leucostachyus* extracts were the most selective (SI >28.2) and the most active in vivo (at doses of 250 mg/kg, 71 % suppression of *P. berghei* parasitaemia versus untreated controls).

**Conclusions:**

Ethnobotanical or ethnopharmacological reports describe the anti-malarial use of these plants or the antiplasmodial activity of congeneric species. No antiplasmodial activity has been demonstrated previously for the extracts of these plants. Seven plants exhibit in vivo and or in vitro anti-malarial potential. Future work should aim to discover the anti-malarial substances present.

## Background

In the Amazon region, the occurrence of malaria is related to demographic, ecological, socio-economic, and cultural changes, especially in the first half of the twentieth century. In this period, relatively few plants from this region were used to treat this disease [1, 2]. Over time, the co-existence of traditional populations with this disease caused a search for new therapeutic resources in the Amazonian environment, especially among plants, to treat the symptoms of malaria. Nowadays, this traditional knowledge, available through ethnopharmacological studies, is the most often used means to target plants for the discovery of new bioactive substances. The ethnopharmacological approach has led to the saving of time and financial resources as compared to other approaches, such as chemosystematic or random plant selection [3, 4]. The chemosystematic approach for the selection of anti-malarial plants for study is also valid especially where ethnopharmacological studies have shown a plant family, or a particular genus, to contain anti-malarial extracts and chemical constituents.

Natural products are the origin of approximately two-thirds of all drugs introduced in the past 30 years [5]. Plants are recognized as important sources of antiprotozoal compounds for the development of drugs against many tropical diseases, including malaria. Examples of anti-malarial natural products are (1) quinine, present in western Amazonian *Cinchona* spp., (2) quassinoids and limonoids in plants of the Simaroubaceae and Meliaceae families, respectively, and, (3) artemisinin from *Artemisia annua*, among others [6, 7].

The Amazon region is megadiverse. Screening for in vitro and in vivo anti-malarial activity of extracts of traditionally used plants from this region is a strategy for the discovery of new anti-malarial substances [8–10]. Studies on the anti-malarial activity of plant species from countries of the Amazon region such as Bolivia [11–15], Brazil [16–29], Colombia [30], French Guiana [31–34], and Peru [35–38] have demonstrated the potential of local traditional medicinal practices as sources of potent extracts and anti-malarial substances.

Krettli and collaborators performed ethnobotanic surveys of anti-malarial plants across the Brazilian Amazon region and applied the ethnopharmacological approach to the study of these plants for the first time [1, 39]. Ethnopharmacology provided a relatively large number (4 in 22 plants or 18 %) of plants exhibiting extracts with in vivo efficacy against *Plasmodium berghei* compared to an approach based on random selection of plants (two active plants in 273 tested or 0.7 %) [40, 41].

In the present work, after a systematic literature search, 11 Amazonian plants were selected based on their use as anti-malarials or based on the proven anti-malarial activity of the plant genus. No previous report on the activity against *Plasmodium* parasites was found for the selected plant species. Their extracts were assayed for in vitro and in vivo anti-malarial activity and cytotoxicity. The aim of this study was to discover Amazonian plant extracts exhibiting important in vitro and in vivo anti-malarial activity as a first step towards bioguided isolation of active principles. The plant species studied are shown in Table 1.

**Table 1:** Information on plant species, voucher specimens, traditional remedies and ethnobotanic sources indicating anti-malarial use

Species	Family	Accession number	Common name	Remedy	Source
*Anacardium occidentale*	Anacardiaceae	INPA 57941	Cajueiro	Bark, leaves, fruit infusions, decoction (10 drops 2×/day of trunk bark alcohol extract)	[58, 68, 82, 96, 109, 113]
*Andropogon leucostachyus*	Poaceae	INPA 250467	Capim-colchão	Whole plant decoction	[58]
*Clidemia bullosa*	Melastomataceae	INPA 250466	Caiuia	Not found^a^	Not found^a^
*Croton cajucara*	Euphorbiaceae	EAFM 315	Sacaca	Bark and leaves infusions	[58, 62–68]
*Derris floribunda*	Fabaceae	INPA 15562	Timbó	Branches	[58]
*Miconia nervosa*	Melastomataceae	INPA 250467	Miraúba	Decoction (part not specified)	[58]
*Parkia nítida*	Fabaceae	INPA 152124	Faveira	Not specified	[58]
*Paullinia cupana*	Sapindaceae	INPA 122001	Guaraná	Leaves, branches, roots, seeds	[58, 63, 96, 97]
*Stigmaphyllon sinuatum*	Malpighiaceae	INPA 205629	Cipó asa de gafanhoto	Leaves decoction	[58]
*Xylopia amazonica*	Annonaceae	INPA 183108	Envira sarassará	Not found^b^	Not found^b^
*Zanthoxylum djalma*-*batistae*	Rutaceae	INPA 210077	Tamanqueira	Not found^c^	Not found^c^

^a^ *Clidemia hirta* is the species cited as being in use by traditional peoples of the Peruvian Amazon [38]

^b^Fruit and trunk bark macerates and infusions of these *Xylopia* spp. are used as anti-malarials: *Xylopia aethiopica*, *Xylopia aromatica*, *Xylopia ​brasiliensis*, *Xylopia emarginata, Xylopia frutescens, Xylopia grandiflora, Xylopia hypolampra, Xylopia longifolia,* *Xylopia parviflora, Xylopia phloiodora, Xylopia staudtii*, *Xylopia xylopioides* [30, 58, 80–85, 125, 126]

^c^These *Zanthoxylum* spp. are used as anti-malarials: *Zanthoxylum armatum*, *Zanthoxylum caribaem, Zanthoxylum chalybeum*, *Zanthoxylum* *chiloperone, Zanthoxylum gilletii*, *Zanthoxylum hermaphroditum, Zanthoxylum leprieurii, Zanthoxylum pentandrum*, *Zanthoxylum* *perrottetti, Zanthoxylum rhoifolium, Zanthoxylum rubescens*, *Zanthoxylum tingoassuiba, Zanthoxylum tsihanimposa*, *Zanthoxylum usambarense,* *Zanthoxylum zanthoxyloides*. Leaf, fruit, trunk bark and root bark decoctions are used [33, 58, 59, 84, 85, 100–105, 107–110, 127–129]

## Methods

### Collection, identification and processing of plant materials

Initially, library and online (Web of Knowledge, Scopus, Scifinder Scholar, among others) surveys of the literature on plants traditionally used as anti-malarials were performed using the convenient search term ‘antimalarials from plants’. Also, a survey of plant species exhibiting proven anti-malarial properties according to previous laboratory studies was performed. Registry (No. 33110-1) for the collection of plant materials was performed online through the Brazilian Government´s Authorization and Information in Biodiversity System (SISBIO), Chico Mendes Biodiversity Conservation Institute (ICMBio), Ministry of Environment (MMA). Collection of the different parts of 11 Amazonian species was performed based on ethnobotanic information, where available. These were generally the more readily collected parts of each plant species (Table 1). Authorization for collection of plant materials at the National Institute for Amazon Research (INPA) Adolpho Ducke Forest Reserve, located in the municipality of Manaus, was obtained prior to collection. Plant materials from Embrapa Amazônia Ocidental´s live plant collections were provided by Dr. Francisco Célio Maia Chaves. No in vitro and/or in vivo anti-malarial activity data were available for these plant species in the literature. Collection was performed from August to October 2012 in Amazonas State, Brazil. Vouchers were deposited and identified at the INPA and Federal Agrotechnical School of Manaus (EAFM) herbariums. Prior to extraction, plant materials were dried in the shade at ambient temperature (average of ca. 27 °C) for 72 h and then further dried in a circulating air oven at 40 °C for 7 days. The dry plant materials were ground and stored at −20 °C until extraction was performed.

### Extraction of plant materials

Ethanol (or methanol), water and chloroform extracts were prepared from each plant part (Table 1). Water extracts were prepared by infusing dry, ground plant materials in boiling de-ionized water (100 °C, 3 × 15 min) or when indicated in the literature, a decoction was prepared by boiling plant materials for 1 h under reflux. Ethanol, chloroform and methanol extractions were performed using a Soxhlet apparatus (3 × 6 h). Extraction solvents were removed by rotary evaporation under vacuum using mild bath temperatures. Crude extracts were transferred to broad-mouthed vials and further evaporated in a 45–50 °C sand bath in a fume hood. After total evaporation, extracts were frozen at −20 °C, freeze-dried and weighed.

### In vitro culture of *Plasmodium falciparum*

Chloroquine-resistant (CQR) *Plasmodium falciparum* W2 clone and K1 strain were used for in vitro antiplasmodial studies. The Trager and Jensen [42] in vitro culture technique was used with modifications [17]. Parasites were cultivated in type A^+^ erythrocytes and culture medium (Roswell Park Memorial Institute or RPMI-1640) enriched with 10 % human serum (complete medium) and maintained at 37 °C under an atmosphere of 5 % carbon dioxide, 5 % oxygen and 90 % nitrogen.

Monitoring of parasite growth was performed every 24 h during the daily refreshing of culture medium. Parasitaemia was calculated as a percentage based on the viable parasitic forms observed by counting at least 2000 erythrocytes.

### Screening for in vitro antiplasmodial activity against *Plasmodium falciparum* K1 strain

Parasite quantification by optical microscopy is a traditional and reliable technique in the performance of in vitro antiplasmodial assays [43]. In vitro antiplasmodial screening was performed according to a previously published procedure [17]. Each extract (1.0 mg) was dissolved in dimethyl sulfoxide (DMSO) to provide a stock solution (5.0 mg/mL). Test solutions of each extract were prepared by diluting stock solution in RPMI-1640 culture medium and 20 μL of each test solution were introduced into the wells of a 96-well plate. Then, a suspension of sorbitol-synchronized, parasitized red blood cells (pRBCs) [44] was adjusted to 1 % parasitaemia and 3 % haematocrit in complete medium and added (180 μL/well). Screening of extracts was performed at final (well) concentrations of 50 and 5.0 μg/mL. Negative controls were prepared with a suspension of pRBCs and 1 % DMSO. Chloroquine was used as positive control. Test plates were incubated at 37 °C for 48 h under an atmosphere of 5 % oxygen, 5 % carbon dioxide and 90 % nitrogen. After this period, thin blood smears of the contents of each well were stained with Panótico^®^ (Laborclin, Pinhais, Paraná, Brazil) for evaluation of the parasitaemia using an optical microscope. The parasitaemia was expressed as a percentage of the viable erythrocytic parasite forms observed in 2000 RBCs. Parasite inhibition was expressed as a percentage of the growth of untreated (negative) controls. All assays were performed in triplicate [45]. Extracts that inhibited parasite growth by ≥80 % at concentrations of 50 μg/mL were further evaluated using the procedure described below.

### In vitro antiplasmodial activity assessed by anti-HRPII ELISA

*Plasmodium falciparum* histidine-rich protein II (HRPII) may be quantified as an indicator of cell growth through the enzyme-linked immunosorbent assay (ELISA)-sandwich technique [46–48]. This technique has been applied to the screening of a variety of crude plant extracts and fractions [49–52] and is a valid method for the screening of plant extracts providing similar IC_50_ values to traditional methods [49, 50].

Extracts exhibiting anti-malarial potential in the screening procedure described above against *P. falciparum* K1 strain were further evaluated to determine the concentrations that inhibit 50 % of parasite growth (IC_50_) using histidine rich protein II antibody (anti-HRPII) ELISA [47] with slight modifications [49]. Briefly, each extract was dissolved in DMSO using an ultrasound bath to form stock solutions (10 mg/mL). Each stock solution was serially diluted in culture medium to provide seven dilute samples. Each dilute sample (20 µL) was applied to a 96-well test plate in triplicate. A suspension of sorbitol-synchronized pRBCs was adjusted to 0.05 % parasitaemia and 1.5 % haematocrit and placed in 96-well plates containing the test and control drugs providing final (in well) extract concentrations of 100–0.13 µg/mL. The plates were incubated for 72 h. After 24 h, the contents of the six control wells (parasites in drug-free medium) were harvested in microtubes and frozen for later use to further exclude the background value (i.e., the production of HRPII during the first 24 h of incubation) by subtracting the average value obtained from these wells from that of the wells containing the test and control drugs. After 72 h of incubation, the plates were frozen and thawed twice to lyse the erythrocytes.

To perform the test, a clean plate (Maxysorp, Nunc, Denmark) was first coated with 100 μL of the primary antibody anti-HRPII (MPFM ICLLAB-55A^®^, Stuart, FL, USA) at 1.0 μg/mL. Following overnight incubation at 4 °C, the antibody solution was discarded and replaced with 200 μL/well of 2 % PBS-BSA (phosphate buffered saline and bovine serum albumin, Sigma-Aldrich) blocking solution. Following a new incubation at room temperature for 2 h, the plate was washed with Tween 20 at 0.05 % in PBS (PBS-T). Then, each pre-treated well received 100 μL of *P. falciparum* parasite culture (as described above), which was pre-haemolyzed by freeze-thawing at −70 °C. In each test, two haemolyzed control sets of six wells each were used; one containing the 24 h cultures (background), the other with the 72 h parasite cultures. After incubation for 1 h at room temperature, the plate was again washed with PBS-T, incubated with 100 μL/well of the secondary antibody (MPFG55P ICLLAB^®^, Stuart, FL, USA), diluted 1:5000 times, and again incubated for 1 h at room temperature. After more washes with PBS-T, each well received 100 μL of 3,3′,5,5′-tetramethylbenzidine (TMB) chromogen (KPL, Gaithersburg, MD, USA) and was incubated for 10 min at room temperature in the dark. The reaction was stopped with 50 μL/well of 1 M sulfuric acid and the absorbance was immediately read at 450 nm on a spectrophotometer (SpectraMax^®^ 340PC384, Molecular Devices, Sunnyvale, CA, USA). Three separate experimental determinations were performed and the average readings were plotted and the IC_50_ values were determined from the plots.

### Screening for in vitro cytotoxicity (cell viability test)

The cytotoxicity of extracts was evaluated in vitro against one non-neoplastic cell line (MRC-5-human fibroblast) purchased from the American Type Culture Collection (ATCC) and two melanoma cell lines (SK-MEL-19 and SK-MEL-28) donated by Dr. María S Soengas (CNIO, Madrid, Spain). The cells were cultivated in 96-well plates (0.5 × 10^4^ cells per well) and then were treated with extract in DMSO at a single concentration (50 μg/mL) over 72 h. The Alamar Blue™ assay was performed after 72 h following a standard procedure [53].

### Murine macrophage culture

J774 cells (murine macrophages) were obtained from the Cell Bank of Rio de Janeiro, Brazil and were maintained in Dulbecco’s Modified Eagle Medium (DMEM), which was supplemented with 10 % fetal bovine serum (FBS), penicillin (100 U/mL) and streptomycin (100 U/mL). The cells were incubated at 37 °C in a humidified atmosphere containing 5 % of CO_2_.

### Determination of in vitro toxicity against murine macrophages (cell viability test)

The cytotoxicity was determined by the Alamar Blue method as described by Nakayama and co-workers [54]. Briefly, adherent cells (5 × 10^3^ cells/well) were grown in 96-well tissue culture plates and exposed to extracts (1.56–200 µg/mL) for 48 h. After incubation, Alamar Blue solution (10 μL of 0.4 % Alamar Blue (resazurin) in water) was added and the cells were incubated for 3 h at 37 °C. Fluorescence was measured (excitation at 545 nm and emission at 595 nm) and expressed as a percentage of the cells in the control after background fluorescence was subtracted. Doxorubicin was used as a positive control of cell death. The assays were performed in triplicate. The IC_50_ values and their 95 % confidence intervals (95 % CI) were obtained by non-linear regression using the Graphpad program (Intuitive software for science, San Diego, CA, USA).

### Animals and ethical approval

Adult BALB/c mice (25 ± 3 g weight) were used for the in vivo anti-malarial tests and received water and food ad libitum. In vivo tests were performed following the Guidelines for Ethical Conduct in The Care and Use of Animals of the National Institute for Amazon Research (INPA). This work was authorized by INPA’s Commission for the Ethical Use of Animals (CEUA 029/2013).

### In vivo anti-malarial test

Three extracts exhibiting in vitro antiplasmodial activity in the above assay were tested for oral activity against the *Plasmodium berghei* NK65 strain in mice with blood-induced infections maintained by successive passages from mouse to mouse. The protocol used was based on the Peters [55] four-day suppressive test with slight modifications [21]. In each experiment, the animals were divided into groups of five individuals and treated for four successive days with extract (250 mg/kg/day) starting 24 h after inoculation with *P. berghei* (1 × 10^5^ parasitized erythrocytes/animal). In each experiment, positive and negative control groups received chloroquine (10 mg/kg/day) and vehicle (2 % DMSO), respectively. Each sample was tested in at least two independent experiments. On days 5 and 7 after infection, blood smears from all mice were prepared, stained with the Panótico^®^ system (Laborclin, Pinhais, Paraná, Brazil) and examined under a microscope. The parasitaemia was determined in blood smears that were characterized by random counting of 2000–4000 erythrocytes when parasitaemia was low (≤10 %) or up to 1000 erythrocytes when parasitaemia was higher. Mortality was monitored daily in all groups during a period of 4 weeks after inoculation.

## Results

### In vitro antiplasmodial activity, cytotoxicity and selectivity of extracts

A total of 69 chloroform, ethanol, methanol, and water extracts from different parts of the 11 plant species were prepared. Screening against *P. falciparum* K1 strain revealed 32 extracts that exhibited antiplasmodial potential (IC_50_ ≤50 μg/mL). These extracts were further evaluated in vitro against the CQR *P. falciparum* W2 clone to establish accurate IC_50_ values. The in vitro IC_50_ values of plant extracts are shown in Table 2. Extracts exhibiting IC_50_ values ≤10 µg/mL were considered to be active. Those exhibiting IC_50_ values in the range 10 to ≤25 μg/mL were considered moderately active. Extracts exhibiting IC_50_ values >25 μg/mL were considered inactive. *Andropogon leucostachyus* aerial part methanol extracts, *Croton cajucara* red variety leaf chloroform extracts, *Miconia nervosa* leaf methanol extracts and *Xylopia amazonica* leaf chloroform and branch ethanol extracts were the most active (7 % of all extracts). Moderate activity (22 % of all extracts) was observed for *Clidemia bullosa* DC branch chloroform extract and decoction, *Croton cajucara* white variety leaf chloroform and ethanol and bark ethanol extracts, *Croton cajucara* red variety leaf ethanol extract, *Miconianervosa* bark and leaf chloroform extracts and leaf decoction, *Paullinia cupana* fruit and branch chloroform extracts, *Xylopiaamazonica* leaf decoction and branch chloroform extract and *Zanthoxylum djalma*-*batistae* leaf decoction and branch chloroform extract. Seventy-one per cent of the extracts were inactive. All extracts prepared from *Anacardium occidentale*, *Derris floribunda*, *Parkia nitida* and *Stigmaphyllon sinuatum* were inactive in vitro against *P. falciparum* as were *Croton cajucara* red variety bark and *Paullina cupana* leaf extracts.

**Table 2:** In vitro median inhibitory concentrations (IC_50_) against *Plasmodium falciparum* strains, toxicity to murine macrophages (IC_50_) and selectivity indices (SI) of plant extracts

Plant species	Part	Extract	*P. falciparum* ^a^ IC_50_, µg/mL ± SD^b^		Macrophages IC_50_, µg/mL (95 % CI)	SI^c^
*Anacardium occidentale*	Bark	CHCl_3_	36.6 ± 17.7	I	>200	>5.5
		EtOH	>50	I	–	–
		H_2_O(i)	>50	I	–	–
	Leaf	CHCl_3_	43.9 ± 10.8	I	>200	>4.6
		EtOH	>50	I	–	–
		H_2_O(i)	45.0 ± 5.0	I	>200	>4.4
*Andropogon leucostachyus*	Aerial part	CHCl_3_	>50	I	–	–
		H_2_O(d)	45.4 ± 0.4	I	>200	>4.4
		MeOH	7.1 ± 3.3	A	>200	>28.2
*Clidemia bullosa*	Leaf	CHCl_3_	>50	I	–	–
		H_2_O(d)	26.2 ± 3.1	I	>200	>7.6
		MeOH	>50	I	–	–
	Branch	CHCl_3_	13.5 ± 2.7	MA	>200	>14.8
		H_2_O(d)	21.2 ± 4.0	MA	–	–
		MeOH	>50	I	–	–
*Croton cajucara* (white variety-WV)	Bark	CHCl_3_	29.1 ± 6.3	I	43.1 (27.4–67.8)	1.5
		EtOH	17.2 ± 6.6	MA	127 (49.8–321)	7.4
		H_2_O(i)	>50	I	–	–
	Leaf	CHCl_3_	11.3 ± 3.4	MA	>200	>17.7
		EtOH	16.3 ± 4.5	MA	>200	>12.3
		H_2_O(i)	>50	I	–	–
*Croton cajucara* (red variety-RV)	Bark	CHCl_3_	32.2 ± 5.7	I	>200	>6.2
		EtOH	>50	I	–	–
		H_2_O(i)	>50	I	–	–
	Leaf	CHCl_3_	6.4 ± 1.2	A	40.6 (32.6–50.6)	6.3
		EtOH	13.3 ± 2.3	MA	>200	>15.0
		H_2_O(i)	>50	I	–	–
*Derris floribunda*	Bark	CHCl_3_	>50	I	–	–
		H_2_O(i)	>50	I	–	–
		MeOH	>50	I	–	–
	Leaf	CHCl_3_	47.4 ± 1.6	I	>200	>4.2
		H_2_O(i)	27.5 ± 7.5	I	–	–
		MeOH	>50	I	–	–
*Miconia nervosa*	Bark	CHCl_3_	13.3 ± 2.0	MA	46.6 (43.1–50.4)	3.5
		H_2_O(d)	>50	I	–	–
		MeOH	>50	I	–	–
	Leaf	CHCl_3_	12.4 ± 4.1	MA	70.6 (62.7–79.7)	5.7
		H_2_O(d)	10.2 ± 2.5	MA	>200	>19.6
		MeOH	9.9 ± 3.2	A	95.9 (71.0–130)	9.7
*Parkia nitida*	Bark	CHCl_3_	>50	I	–	–
		H_2_O(d)	>50	I	–	–
		MeOH	>50	I	–	–
	Leaf	CHCl_3_	>50	I	–	–
		H_2_O(d)	>50	I	–	–
		MeOH	>50	I	–	–
*Paullinia cupana*	Leaf	CHCl_3_	>50	I	–	–
		H_2_O(i)	>50	I	–	–
		MeOH	>50	I	–	–
	Fruit	CHCl_3_	19.3 ± 6.4	MA	>200	>10.4
		H_2_O(i)	>50	I	–	–
		MeOH	>50	I	–	–
	Branch	CHCl_3_	19.3 ± 5.5	MA	62.9 (53.9–73.4)	3.3
		H_2_O(i)	>50	I	–	–
		MeOH	>50	I	–	–
*Stigmaphyllon sinuatum*	Leaf	CHCl_3_	>50	I	–	–
		EtOH	>50	I	–	–
		H_2_O(i)	>50	I	–	–
*Xylopia amazonica*	Leaf	CHCl_3_	7.3 ± 1.8	A	33.9 (29.6–38.9)	4.6
		H_2_O(d)	10.5 ± 3.3	MA	>200	>19.0
		EtOH	>50	I	–	–
	Branch	CHCl_3_	19.5 ± 3.1	MA	29.2 (19.6–43.5)	1.5
		H_2_O(d)	>50	I	–	–
		EtOH	9.8 ± 1.8	A	6.9 (0.4–12.1)	0.7
*Zanthoxylum djalma*-*batistae*	Leaf	CHCl_3_	40.2 ± 3.2	I	>200	>5.0
		H_2_O(i)	15.6 ± 2.9	MA	>200	>12.8
		MeOH	>50	I	–	–
	Branch	CHCl_3_	17.4 ± 1.3	MA	24.7 (18.6–32.9)	1.4
		H_2_O(i)	32.5 ± 7.9	I	>200	>6.2
		MeOH	21.8 ± 3.7	I	>200	>9.2
Controls	DMSO		–	I	–	–
	Chloroquine diphosphate		0.23 ± 0.03	A	–	–
	Doxorubicin		–	–	0.63 (0.59–0.68)	–

*EtOH* ethanol, *MeOH* methanol, *H*_*2*_ *O*(*i*) infusion, *H*_*2*_ *O*(*d*) decoction, *SD* standard deviation, *95* *% CI* 95 % confidence interval,  – not evaluated, not determined

^a^All extracts were screened at 50 and 5 mg/mL against *P. falciparum* K1 strain using optical microscopy in three separate experiments. Accurate IC_50_ values were determined using seven concentrations of extract against *P. falciparum* W2 strain using the HRP2-ELISA method

^b^Antiplasmodial effect based on IC_50_: *A* active (IC_50_ ≤ 10 µg/mL), *MA* moderately active (10 < IC_50_ ≤ 25 μg/mL) and *I* inactive (IC_50_ > 25 μg/mL)

^c^SI = IC_50(murine macrophages)_/IC_50(*P. falciparum*)_

During initial screening, those extracts that reduced in vitro cell viability to less than 10 % of that of untreated controls after 72 h were considered to be cytotoxic. Thus, only *Paullina cupana* branch chloroform extract was toxic to MRC-5 cells (6.8 % viability after 72 h). This same extract was toxic to SK-MEL-19 and SK-MEL-28 melanoma cells (7.3 and 6.6 % viability, respectively). *Croton cajucara* red variety leaf ethanol extract was also toxic to SK-MEL-28 cells. All other extracts did not significantly inhibit proliferation of MRC-5, SK-MEL-19 or SK-MEL-28 cells (viability >50 % at concentrations of 50 μg/mL).

Thirty extracts were evaluated for in vitro toxicity against murine macrophages to establish IC_50_ values and selectivity parameters (Table 2). Twenty-three extracts were essentially non-toxic to murine macrophages (IC_50_ values >50 μg/mL). However, *Xylopia amazonica* branch ethanol and *Zanthoxylum djalma*-*batistae* branch chloroform extracts (IC_50_ = 6.9 and 24.7 μg/mL, respectively) exhibited significant cytotoxicity (IC_50_ <25 μg/mL) to macrophages. *Andropogon leucostachyus* aerial part methanol extracts exhibited the greatest selectivity index (SI >28.2) (Table 2).

### In vivo activity of extracts

Three extracts exhibiting in vitro antiplasmodial activity were assayed for in vivo anti-malarial activity and the results are presented in Table 3. *Andropogon leucostachyus* extract was the most active in vivo exhibiting 71 % suppression of *P. berghei* parasitaemia on the fifth day. *Xylopia amazonica* leaf extracts exhibited 52 % suppression on the fifth day of infection and low in vivo parasite suppression was observed for *Croton cajucara* red variety leaf extracts (Table 3).

**Table 3:** Parasitemia suppression versus untreated controls and survival in mice infected with *Plasmodium berghei* after oral administration of plant extracts for four days

Plant	Part	Extract	Dose (mg/kg/day)	% parasite ± SD (% suppression)		Avg. survival ± SD (days)
				Day 5	Day 7	
*Andropogon leucostachyus*	Aerial part	MeOH	250	0.49 ± 0.10 (71)	1.12 ± 0.07 (48)	19 ± 2
*Croton cajucara* RV	Leaf	CHCl_3_	250	1.4 ± 0.27 (19)	2.2 ± 0.15 (0)	23 ± 3
*Xylopia amazonica*	Leaf	CHCl_3_	250	0.82 ± 0.22 (52)	1.87 ± 0.26 (11)	20 ± 2
Controls		Chloroquine diphosphate	10	0.15 ± 0.04 (91)	0.14 ± 0.04 (93)	31 ± 4
		Vehicle (Blank)		1.74 ± 0.21	2.12 ± 0.31	22 ± 1

*CHCl*_*3*_ chloroform, *MeOH* methanol, *SD* standard deviation

## Discussion

Several strategies are available for the discovery of new anti-malarial drugs. In vitro screening for inhibitory activity against *P. falciparum* and identification of traditionally used plant extracts exhibiting IC_50_ values less than 10 µg/mL are important first steps in the search for new anti-malarial plant extracts. Similar approaches have led to the identification of extracts for chemical composition studies and the discovery of potent plant natural products, such as artemisinin and nimbolide [56, 57].

In traditional medicine, *Andropogonleucostachyus* whole plant decoctions are ingested as a treatment for malaria [58]. In this work, *Andropogon leucostachyus* aerial part decoctions exhibited low in vitro activity. Methanol extraction was the most efficient process for concentrating the in vitro (IC_50_ = 7.1 ± 3.3 µg/mL against *P. falciparum*) anti-malarial activity and selectivity of *Andropogon leucostachyus*. In fact, *Andropogon leucostachyus* aerial part methanol extracts exhibited the highest selectivity index (SI = 28.2) of all extracts evaluated herein. The concentration of anti-malarial components in these extracts is further attested to by the in vivo result (71 % suppression of *P. berghei*). Interestingly, the leaf decoctions of a related species, *Andropogon schoenanthus*, are ingested (with large amounts of sugar) to treat malaria fevers. Inhalation of the vapours from the boiling decoction is also used to treat malaria [59]. Very little is known about the chemical composition of *Andropogon leucostachyus*. *C*-glycosylflavones, the *O*-methyl flavone tricin and the flavanol luteoforol have been described in the leaves of *Andropogon leucostachyus* [60]. No anti-malarial activity has been reported for these flavonoids in the literature. In silico docking studies have explored the potential of tricin as a parasite dihydrofolate reductase inhibitor however it was found to interact less favorably with this enzyme than other compounds [61].

*Croton cajucara* is a cultivated plant that has red and white varieties (a reference to the coloration of young leaves). It occurs in Bolivia, Brazil, Guyana and Venezuela. *Croton cajucara* trunk bark or leaf infusions are used in traditional medicine to treat malaria according to many sources [62–68]. Herein, *Croton cajucara* extracts of both varieties were active or moderately active in vitro. Red variety leaf chloroform extract exhibited the highest in vitro inhibitory activity against *P. falciparum* W2 clone (IC_50_ = 6.4 ± 1.2 µg/mL). This extract was further evaluated for in vivo oral activity against *P. berghei* in infected mice, however, it exhibited low in vivo anti-malarial activity (Table 3). Synergism among the bioactive components that comprise an extract could explain in vitro antiplasmodial activity however in vivo these chemical constituents may have a lessened effect due to their metabolism (biotransformation), low bioavailability and physiological factors in the host [69].

A number of *Croton* species have been found in previous studies to exhibit significant in vitro and in vivo anti-malarial activity. *Croton leptostachyus* aerial part ethanol extracts exhibited high in vitro activity against *P. falciparum* (IC_50_ = 2.1 ± 0.2 µg/mL), however, this extract was toxic to mice [30]. Several *Croton zambesicus* root extracts and fractions exhibited in vivo anti-malarial activity (79–86 % parasitaemia suppression at doses of 27–81 mg/kg/day) against *P. berghei* in rodents [70]. Also, *Croton mubango* stem bark water extracts inhibited *P. falciparum* in vitro (IC_50_ = 3.2 µg/mL) and suppressed *P. berghei* ANKA by 77 % at oral doses of 200 mg/kg/day [71]. Significant dose dependency in the suppression of *P. berghei* in mice has been observed for *Croton macrostachyus* water and methanol extracts (200, 400 and 600 mg/kg) [72]. Similar results were obtained for crude extracts and chloroform, methanol and water fractions of this same species wherein the chloroform fraction exhibited the best result [73].

A number of antiplasmodial diterpenes have been isolated from *Croton* species. 8,9-secokaurane was isolated from *Croton kongensis* and inhibited *P. falciparum* K1 strain (IC_50_ = 1–2.8 µg/mL) [74] and geranyl geraniol was isolated from *Croton lobatus* extracts and inhibited *P. falciparum* (IC_50_ = 3.7 µM) [64]. Steenkrotin A, was isolated from *Croton steenkampianus* leaf ethanol extracts and exhibited IC_50_ = 15.8, >30, 9.4 and 9.1 µM against *P. falciparum* D10, D6, Dd2 and W2 clones, respectively [66].

*Miconia nervosa* is used traditionally in the treatment of malaria as a decoction as are *Miconia laevigata* and *Miconia willdenowii* [58, 62, 75, 76]. Herein, *Miconia nervosa* leaf extracts exhibited in vitro activity against *P. falciparum* W2 clone. No previous report on the antiplasmodial activity of extracts of a species of *Miconia* is available in the literature. Interestingly, other species from this genus, *Miconia fallax* and *Miconia stenostachya*, are known to produce triterpene compounds that inhibit the protozoa *Trypanosoma cruzi* [77].

*Xylopia amazonica* was revealed herein as a plant whose crude extracts have anti-malarial potential. Two of its extracts were active and a third was moderately active. Among these, the leaf chloroform extracts exhibited good in vitro antiplasmodial activity (IC_50_ = 7.3 µg/mL) and no significant toxicity to human fibroblasts or melanoma cells was observed. Notwithstanding, these extracts exhibited low selectivity (SI = 4.6), which is an indication that chloroform extraction concentrates specific toxicity to *P. falciparum* and murine macrophages. Interestingly, cytotoxicity has been observed for the extracts of *Xylopia aromatica* trunk chloroform–methanol extracts against NCI-H460, KM-12 and SF-268 cell lines and cancer cell line RPMI-8226 [78]. Also, *Xylopia aromatica* wood hexane extracts exhibited IC_50_ = 5–20 µg/mL against several tumour cell lines (SF-295, HCT-8, MDA-MB-435 and HL-60) [79].

In several countries, the macerated or infused fruit and/or trunk bark of at least a dozen *Xylopia* species are used to treat malaria [30, 58, 80–85]. The extracts of several of these plants exhibit in vitro antiplasmodial activity according to previous studies. *Xylopia phloiodora* and *Xylopia aethiopica* extracts inhibit *P. falciparum* in vitro (IC_50_ = 18 µg/mL) [80]. *Xylopia emarginata* leaf [81], root bark, trunk bark and wood [86] extracts exhibit IC_50_ = 3–11 µg/mL against *P. falciparum* Palo Alto or FcB1 strains. The ethanol extract of the aerial parts of *Xylopia aromatica* strongly inhibit *P. falciparum* in vitro (IC_50_ <1 µg/mL) [30] while root, root bark and trunk bark hexane extracts do so to a lesser extent (IC_50_ = 4.7, 6.8 and 15.3 µg/mL, respectively) against *P. falciparum* FcB1 strain [86].

Besides the in vitro antiplasmodial activity observed, *Xylopia amazonica* leaf chloroform extracts administered orally were able to suppress (52 %) *P. berghei* in mice at daily doses of 250 mg/kg herein. For a related species, *Xylopia aromatica*, it was found that aerial part ethanol extracts strongly inhibited *P. falciparum* in vitro (IC_50_ <1 µg/mL) but were inactive in vivo [30].

*Xylopia amazonica* is known to produce kaurene diterpenes and aporphine alkaloids. From the wood and or bark, the diterpenes beyerene, *ent*-kauran-16β-ol, *ent*-kaur-16-en-19-oic acid (kaurenoic acid) and 4-*epi*-kaurenoic acid have been isolated [87, 88] as have the aporphine alkaloids liriodenine, dicentrinone [87], oxoglaucine, (+)-glaucine, lirioferine and (+)-laurotetanine [87, 88].

Importantly, several of the natural products isolated from *Xylopia amazonica* have been isolated from other plant species and found to exhibit in vitro antiplasmodial activity. Thus, (+)-laurotetanine (isolated from *Nectandra salicifolia*) exhibited IC_50_ values of 3.9 and 2.5 μg/mL against *P. falciparum* D6 and W2 parasite clones [89]. Dicentrinone from another species inhibited *P. falciparum* K1 strain (IC_50_ = 1.2 μg/mL) [90]. Liriodenine inhibited *P. falciparum* D6, D10 and W2 clones (IC_50_ = 1.3, 4.1 and 2.4 μg/mL, respectively) [91, 92] whereas oxoglaucine exhibited low activity [92]. The diterpene *ent*-kaur-16-en-19-oic acid was not active against chloroquine-sensitive *P. falciparum* D10 clone (IC_50_ = 31.8 μg/mL) [93].

*Clidemia hirta* known as soap bush or Koster’s curse, is used as an anti-malarial among the traditional peoples of the Peruvian Amazon [38]. Herein, three *Clidemia bullosa* extracts exhibited moderate in vitro antiplasmodial activity. There is no information referring specifically to the traditional anti-malarial use of this species. However, *Clidemia bullosa*, *Clidemia hirta* and another species are closely related and occur together [94]. No information is available on the chemical composition of *Clidemia bullosa*, however, recent work on the chemical composition of the related species, *Clidemia hirta*, revealed the presence of hydrolysable tannins, derivatives of ellagic acid and the triterpene arjunolic acid [95].

Most *Paullinia cupana* (guaraná) extracts did not exhibit antiplasmodial activity. Bark and fruit chloroform extracts of this plant exhibited only moderate antiplasmodial activity. Guaraná extracts are widely consumed in the form of soft drinks and other beverages in Brazil. This plant is used in anti-malarial remedies in different locations in Latin America [58, 63, 96, 97]. In the branch bark, catechin and epicatechin have been detected [98]. These compounds may contribute, together with other compounds, to the moderate antiplasmodial properties observed [99].

*Zanthoxylum djalma*-*batistae* leaf infusion and branch chloroform extracts exhibited moderate antiplasmodial activity (IC_50_ = 15.6 ± 2.9 and 17.4 ± 1.3 µg/mL, respectively) herein. More than a dozen *Zanthoxylum* species are reported to be used as anti-malarials in several countries [33, 59, 84, 85, 100–110] and antiplasmodial activity has been observed for extracts of these plants. Thus, *Zanthoxylumchalybeum* extracts inhibit *P. falciparum* in vitro (IC_50_ <10 μg/mL) [100, 102, 104, 107]. Also, *Zanthoxylumusambarense* trunk bark and trunk wood methanol extracts inhibit *P. falciparum* NF54 strain (IC_50_ <5 µg/mL) [103].

There is no information on the chemical composition of *Zanthoxylumdjalma*-*batistae*. Antiplasmodial benzophenanthridine alkaloids such as nitidine are found in *Zanthoxylum* and other Rutaceae species. Nitidine has been isolated from *Zanthoxylumusambarense* and *Zanthoxylum rhoifolium* [33, 111] and exhibits submicromolar IC_50_ values against *P. falciparum* [33, 112].

Use of cashew tree (*Anacardium occidentale*) trunk bark, leaves or fruit in the treatment of malaria symptoms is practiced by traditional peoples in Brazil, Colombia, Nigeria and Peru [58, 68, 82, 96, 113]. *Anacardium occidentale* leaves are reported to contain anacardic acid and cardol [58, 114]. Anacardic acid is believed to alter parasite gene expression and inhibit parasite development in vitro through enzyme inhibition. In vitro this compound was inactive (IC_50_ = 30.4–34.8 µM) against a number of *P. falciparum* strains (3D7, D10, 7G8 and Dd2) [115]. *Anacardium occidentale* extracts were in general inactive in the present study.

Surprisingly, *Derris floribunda* extracts were inactive herein despite the traditional use of timbó as an anti-malarial and previous reports on the antiplasmodial activity of *Derris* species. *Derris amazonica* extracts have been previously shown to inhibit *P. falciparum* F32 strain in vitro (IC_50_ = 3.2 µg/mL) [15] and lupifolin has been isolated from *Derris trifoliata* seed pod extracts and inhibits *P. falciparum* D6 and W2 strains (IC_50_ = 2.6–3.7 µg/mL) [116].

In animal models of malaria, large experimental variability of the results is associated with drug, parasite and host interactions. For ethical reasons, the numbers of animals used may not be increased to more accurately characterize the antiparasitic effects. Despite this low experimental reproducibility, the murine malaria model used herein is an important tool in anti-malarial drug discovery and development programmes.

Methods and criteria vary among research groups that investigate the anti-malarial potential of plants using rodent models. Extract doses of 300–650 mg/kg/day providing 47.0–84.5 % parasitaemia suppressions have been considered evidence of important anti-malarial activity [22, 117–119]. Also, extracts providing >60 % suppression of parasitaemia at oral doses of 100–250 mg/kg/day in the rodent model have been deemed active or highly active and suppression >30 % at these doses has been deemed moderate activity [120–123]. In the present work, oral doses of 250 mg of plant extract per kg of body weight per day were used for evaluation of plant extracts and detection of relevant parasitaemia suppression (>30 %) on the fifth and or seventh days in the rodent model.

Herein, significant in vivo oral suppression of *P. berghei* by *Andropogon leucostachyus* and *Xylopia amazonica* extracts was demonstrated. Significant differences were not observed in the mean survival of animals treated with these extracts and untreated controls. This is true even for *Andropogon leucostachyus* extracts that were responsible for the largest suppression of parasitaemia observed. In the antiplasmodial extracts tested in vivo, the substances responsible for the suppressive activity may be present in low amounts and may exhibit short half-lives, thus not attaining the concentrations necessary for parasite suppression after the end of treatment. This together with rapid parasite metabolism may provide extract-treated and control groups exhibiting equal parasitaemia after only a few parasitic cycles, which for *P. berghei* is 24 h [124].

The in vitro inhibition of *P. falciparum* and selectivity demonstrated by several plant extracts and oral suppression of *P. berghei* by *Andropogon leucostachyus* and *Xylopia amazonica* extracts are significant findings. Bioguided fractionation of several of the extracts revealed in this study is now underway and should reveal the anti-malarial chemical constituents of these plant species in the future.

## Conclusions

Anti-malarial plants used traditionally in the Amazon and closely related species should be investigated for their anti-malarial potential to increase the knowledge of the useful flora of this region and provide active extracts. The identification of antiplasmodial and cytotoxic, traditionally used species can be useful as an initial step in pharmacological evaluations that can lead to more rational use. Furthermore, antiplasmodial plant extracts are the starting point for bioguided isolation of new anti-malarial chemical constituents. It is always important to remember that among the clinically most relevant anti-malarials in use today are the synthetic quinolines (quinine analogues) and semi-synthetic artemisinin derivatives that comprise artemisinin-based combination therapy (ACT). Both of these classes of anti-malarials owe their origins to quinine and artemisinin discovered in traditionally used plants from the Peruvian Amazon region and China, respectively. Herein, 29 % of the extracts studied were active or moderately active in vitro. The choice of plants studied was based on the traditional use of the species (ethnobotanic approach) or on the traditional use and or proven anti-malarial activity of plants and chemical constituents from the same genus (chemosystematic approach). The large number of active extracts attests to the importance of the accumulated traditional knowledge of anti-malarial plants in the Amazon region. The toxicity of a few extracts to tumour and non-tumour cells should serve as an alert that further toxicological evaluation of these plant extracts is necessary. The few extracts evaluated for in vivo anti-malarial activity were those that exhibited optimal in vitro antiplasmodial activity. However, in vivo evaluation of extracts that correspond to traditional plant remedies, generally infusions, decoctions and tinctures, and fractions enriched in anti-malarial components is necessary as a means to more fully evaluate their anti-malarial potential. Bioguided chemical studies on the active extracts of *Andropogon leucostachyus, Croton cajucara* and *Xylopia amazonica* are now underway and should reveal the antiplasmodial components of these plants in the future.
